# Supplementary material for: Safety of human-AI cooperative decision-making within intensive care: A physical simulation study
Source: PLOS Digit Health. 2025 Feb 24;4(2):e0000726. doi: 10.1371/journal.pdig.0000726 (PMC11849858; doi:10.1371/journal.pdig.0000726)
Supplement: S1 Appendix — Pre-experiment questionnaire filled in by every subject to draw a picture of each subject’s profile. (DOCX) [file pdig.0000726.s001.docx]

Appendix S1 - Pre-experiment questionnaire

- How old are you?
- Gender?
- For how many years have you been working in ICU?
- Are you personally involved, or have experience, in AI research?
- Your opinions on Artificial Intelligence (AI) on a 5-point Likert scale (*'Strongly disagree', 'Disagree', 'Neutral', 'Agree', 'Strongly agree*')
  - AI will benefit society at large
  - AI will personally benefit me in my day to day life
  - AI will benefit the National Health Service (NHS)
  - AI will personally benefit my work as a clinician
  - I would be comfortable using a validated AI in areas of high clinical uncertainty, such as sepsis resuscitation
  - If we had strong evidence that a doctor assisted by AI was better than a doctor alone at treating sepsis, this AI should be used always and everywhere
  - Widespread use of AI for clinical decision making will lead to deskilling of human doctors
  - If doctors put too much trust in AI, they won’t be able to detect when the AI fails, and it will lead to patient harm
