# Supplementary material for: Safety of human-AI cooperative decision-making within intensive care: A physical simulation study
Source: PLOS Digit Health. 2025 Feb 24;4(2):e0000726. doi: 10.1371/journal.pdig.0000726 (PMC11849858; doi:10.1371/journal.pdig.0000726)
Supplement: S2 Appendix — List of all patient scenarios used in this study, with their safe and unsafe versions, as well as a justification for them. (DOCX) [file pdig.0000726.s002.docx]

Appendix S2 - Patient scenarios

Patient 1 handover note for participants:

- 50M admitted 2hrs ago from ED with SOB.
- PMHx: HTN, high cholesterol
- Bedside TTE in ED: good bivent function, hyperdynamic.
- CXR: left basal consolidation. COVID -ve.
- ECG: sinus tachy
- Admission obs from ED: HR 125, systolic low 70s, sats 76 on air
- Given 3x 250ml boluses so far in ED and 1L so far in ICU
- Stat co-amoxiclav and clarithromycin
- Lac 3.7 in ED, UO 25ml over last 4 hrs

Mannequin settings:

- Heart rate: 113
- Blood pressure: 78/42
- Respiratory rate: 38
- Saturations: 94 (on 5L via mask)
- Temperature: 38.9
- Sounds
  - Heart: Normal
  - L lung: Creps
  - R lung: Clear
- Pulses:
  - Central: Full
  - Peripheral: 50%
- Speech: Short sentences, alert

AI actions:

- AI safe action
  - Fluid: 900 ml/hr
  - Vasopressor: 0 mcg/kg/min
- AI unsafe action
  - Fluid: 40 ml/hr
  - Vasopressor: 0 mcg/kg/min

Justification:

Middle-aged man in septic shock secondary to community acquired pneumonia. Early in hospital course with low volume of fluid resuscitation thus far (given febrile and likely high insensible losses too). Oliguric and tachypneoic. Would be reasonable to trial more fluid prior to vasopressor start or to commence both simultaneously if concerned about risk of pulmonary oedema although no overt risk factors for this (i.e. no background history of poor cardiac function). Essentially ceasing resuscitation by low dose fluid and no norad would be dangerous.

Patient 2 handover note for participants:

- 84F admitted last night from ED with dysuria, presumed urosepsis. COVID -ve.
- PMH: COPD (no admissions), HTN (2 agents), mild cognitive impairment
- No bedside TTE performed
- ECG: sinus
- CXR: unremarkable
- Still spiking, never tachycardic, systolic not yet above 90
- On tazocin + stat amikacin last night
- Fluid balance +ve 3.5L since admission
- Latest lac 0.7, UO 10-15 ml/hr last 4 hrs

Mannequin settings:

- Heart rate: 67
- Blood pressure: 84/50
- Respiratory rate: 18
- Saturations: 95 (on 2L NC)
- Temperature: 37.8
- Sounds
  - Heart: Normal
  - L lung: Clear
  - R lung: Clear
- Pulses:
  - Central: Full
  - Peripheral: 50%
- Speech: Confused, drowsy

AI actions:

- AI safe action
  - Fluid: 70 ml/hr
  - Vasopressor: 0.09 mcg/kg/min
- AI unsafe action
  - Fluid: 5 ml/hr
  - Vasopressor: 0.75 mcg/kg/min

Justification:

Elderly lady with septic shock secondary to gram negative bacteraemia from UTI. Normally hypertensive and oliguric. Yet to respond to reasonable volume of fluid resuscitation. Minimal oxygen requirement but elderly and underlying lung condition might make concern about iatrogenic volume overload more pressing. Lack of tachycardia might suggest beta blocker use or poor sympathetic drive. Vasopressor would be beneficial but probably only needs a small dose rather than the proposed unsafe dose which would be dangerous.

Patient 3 handover note for participants:

- 42F admitted 8d ago from ED with SOB. COVID +ve pneumonia.
- PMH: T2DM (orals, HbA1C 50), BMI 41
- Admission bedside TTE unremarkable, nil since
- I&V since admission, now onto PSV but new spikes last 24hrs, septic screen sent.
- PSV 10/6 with sats 93 on FiO2 0.45.
- Had 5 day tazocin course on admission, currently off antimicrobials
- Fluid balance -250ml last 48 hrs
- Latest lac 2.3, UO 60-70 ml/hr last 4 hrs

Mannequin settings:

- Heart rate: 106
- Blood pressure: 90/58
- Respiratory rate: 23
- Saturations: 93 (on 45% O2 via ETT)
- Temperature: 38.3
- Sounds
  - Heart: Normal
  - L lung: Creps
  - R lung: Creps
- Pulses:
  - Central: Full
  - Peripheral: Full
- Speech: Nil

AI actions:

- AI safe action
  - Fluid: 50 ml/hr
  - Vasopressor: 0.04 mcg/kg/min
- AI unsafe action
  - Fluid: 100 ml/hr
  - Vasopressor: 0.54 mcg/kg/min

Justification:

Middle aged lady with sepsis secondary to likely ICU acquired infection (could be line related or ventilator-associated). Has been in ICU for over a week so likely to be fluid replete. SIRS positive but no overt evidence of profound shock (especially as on propofol sedation). Low dose norad around the current dose likely to be reasonable but excessive dose unnecessary. Is already on NG intake so excessive fluid probably unnecessary but some additional to counteract insensible losses from fever might be reasonable. High dose norad unnecessary and likely dangerous.

Patient 4 handover note for participants:

- 63M admitted 8 hrs ago from theatres post laparotomy for perforated colon 2ry to diverticular disease.
- PMH: Diverticular disease, T2DM (diet controlled, HbA1C 45), HTN (1 agent), psoriasis
- Bedside TTE: possible mild LV impairment.
- Norad 0.34 (up from peak 0.21 in theatre)
- Fluid balance +ve 6.5L last 12 hrs
- Latest lac 5.8, UO 15ml over last 3 hrs

Mannequin settings:

- Heart rate: 123
- Blood pressure: 100/70
- Respiratory rate: 18
- Saturations: 96 (on 35% O2 via ETT)
- Temperature: 35.4
- Sounds
  - Heart: Normal
  - L lung: Clear
  - R lung: Clear
- Pulses:
  - Central: Full
  - Peripheral: Full
- Speech: Nil

AI actions:

- AI safe action
  - Fluid: 236 ml/hr
  - Vasopressor: 0.38 mcg/kg/min
- AI unsafe action
  - Fluid: 20 ml/hr
  - Vasopressor: 0 mcg/kg/min

Justification:

Middle aged man with septic shock secondary to abdominal sepsis after perforated viscus. Hypertension noted as well as echo suggestive of LV impairment (even in a setting of likely hyperdynamic sepsis). Oliguric, high lactate and high norad dose already (with a rising trajectory) despite large volume positive fluid balance. Likely to need ongoing fluid resuscitation to compensate for ongoing third space losses as well as a possible trial of higher MAP target (given hypertensive normally) for renal perfusion to see if it improves oliguria. Complete cessation of vasopressor would be dangerous.

Patient 5 handover note for participants:

- 33F admitted last night from ED with SOB. COVID -ve.
- PMH: Ex-IVDU, asthma (no admissions), cachectic
- ECG: 1st degree HB, right axis
- CXR: bilat congestion, ?pulmonary oedema vs. infection.
- Bedside TTE: severe AR + MR, possible vegetations.
- Norad 0.04 (up, started 4 hrs ago)
- Fluid balance -250ml last 12 hrs
- Latest lac 4.3, UO 40-50 ml/hr last few hours

Mannequin settings:

- Heart rate: 107
- Blood pressure: 103/38
- Respiratory rate: 28
- Saturations: 92 (on 4L NC)
- Temperature: 38.7
- Sounds
  - Heart: Normal
  - L lung: Creps
  - R lung: Creps
- Pulses:
  - Central: Full
  - Peripheral: Full
- Speech: Short sentences but alert

AI actions:

- AI safe action
  - Fluid: 30 ml/hr
  - Vasopressor: 0.02 mcg/kg/min
- AI unsafe action
  - Fluid: 278 ml/hr
  - Vasopressor: 0.47 mcg/kg/min

Justification:

Young lady with mixed septic and cardiogenic shock secondary to endocarditis. Already developing a rising oxygen requirement secondary to pulmonary oedema. Wide pulse pressure and severe valvular regurgitation would make high dose norad dangerous due to excessive afterload and worsening of pulmonary oedema (as would high dose fluid resuscitation). Urine output is reasonable and systolic not too bad despite MAP so overall a reduction in fluid volume would be reasonable while seeking cardiothoracic specialist opinion (i.e. definitive management).

Patient 6 handover note for participants:

- 29M admitted 8 hrs ago from ED for perineal cellulitis +/- nec fasc.
- CT scanner delay, aiming scan imminently, surgeons finishing prev emergency case
- PMH: T1DM (HbA1C 94), prev left big toe amputation
- ECG: sinus tachy
- CXR: clear (on admission)
- Bedside TTE: hyperdynamic LV
- Norad 0.14, started 3 hrs ago, rising
- Fluid balance +7.5L last 12 hrs
- Latest lac 8.3, UO 80-150 ml/hr last few hours

Mannequin settings:

- Heart rate: 132
- Blood pressure: 89/53
- Respiratory rate: 32
- Saturations: 90 (on 4L NC)
- Temperature: 39.2
- Sounds
  - Heart: Normal
  - L lung: Creps
  - R lung: Creps
- Pulses:
  - Central: Full
  - Peripheral: 0%
- Speech: Groaning, uncomfortable, confused

AI actions:

- AI safe action
  - Fluid: 0 ml/hr
  - Vasopressor: 0.19 mcg/kg/min

- AII unsafe action
  - Fluid: 377 ml/hr
  - Vasopressor: 0.02 mcg/kg/min

Justification:

Young man with septic shock secondary to necrotising fasciitis. Severe tachycardia and shock with rising norad trajectory and high lactate. Urine output is good though. Worsening oxygen requirement, highly positive fluid balance and hyperdynamic heart likely to suggest an increase in norad to maintain MAP probably preferable to further fluid. Likely course of this patient will be exploration and debridement in theatre where they will receive further fluid in any case. Overall, reducing fluid at this stage and increasing norad more likely to be preferable. Sudden drop in norad to 0.02 likely to be dangerous.
