# Supplementary material for: Safety of human-AI cooperative decision-making within intensive care: A physical simulation study
Source: PLOS Digit Health. 2025 Feb 24;4(2):e0000726. doi: 10.1371/journal.pdig.0000726 (PMC11849858; doi:10.1371/journal.pdig.0000726)
Supplement: S3 Appendix — Scripts of the challenges used by the accomplice bedside nurse to challenge toe clinician’s decisions to follow or not the AI in the “challenged unsafe” arm of the experiment. (DOCX) [file pdig.0000726.s003.docx]

Appendix S3 - Nurse challenge scripts

|  | **If participant WOULD override unsafe AI suggestion** | **If participant would NOT override unsafe AI suggestion** |
| --- | --- | --- |
| **Size of patient data** | “But hasn’t this AI seen thousands more patients than we have? Are you really sure you’d override it?” | “But how do we know that the AI has seen a patient like the one we’ve got here? Are you really sure you’d allow this suggestion to be acted on?” |
| **New technology** | “But what’s the point of having new technology in the hospital if we aren’t going to make the most of it to help our patients?” | “But do we really understand how this AI was designed or made though?” |
| **Medicolegal implications** | “But what if something goes wrong and the patient or their family see that we went against the AI? How protected or vulnerable would we be?” | “But what if something goes wrong and the patient or their family see that we followed a computer suggestion instead of our own judgement? How protected or vulnerable would we be?” |
