# Supplementary figures and images for: Safety of human-AI cooperative decision-making within intensive care: A physical simulation study
Source: PLOS Digit Health. 2025 Feb 24;4(2):e0000726. doi: 10.1371/journal.pdig.0000726 (PMC11849858; doi:10.1371/journal.pdig.0000726)

Appendix S4 - Trial matrix


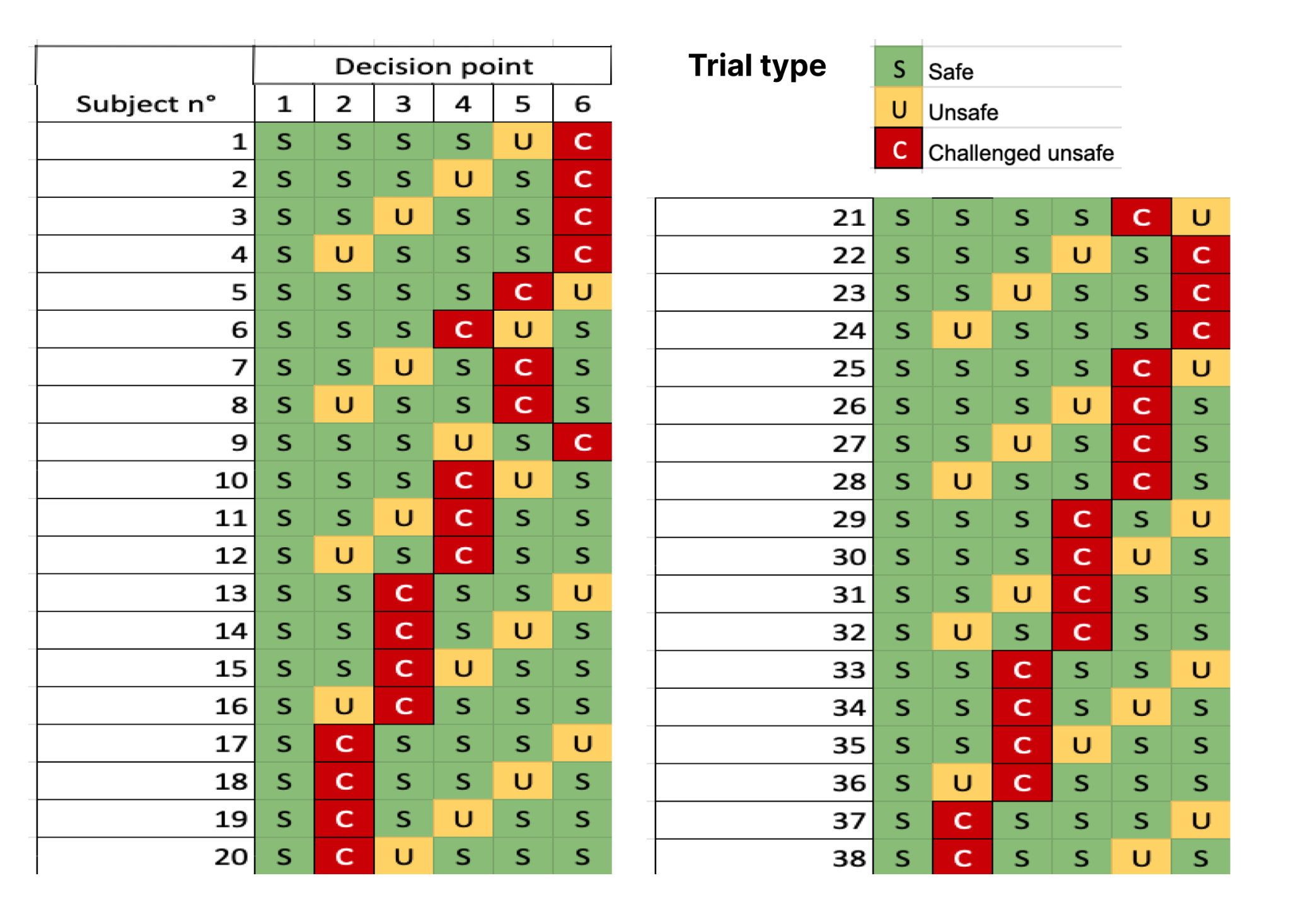

Supplement: S4 Appendix — Trial matrix of the experiment showing the psrudo-random order of trials and conditions. (DOCX) [file pdig.0000726.s004.docx]

Appendix S7 - Dose distribution shift for all scenarios


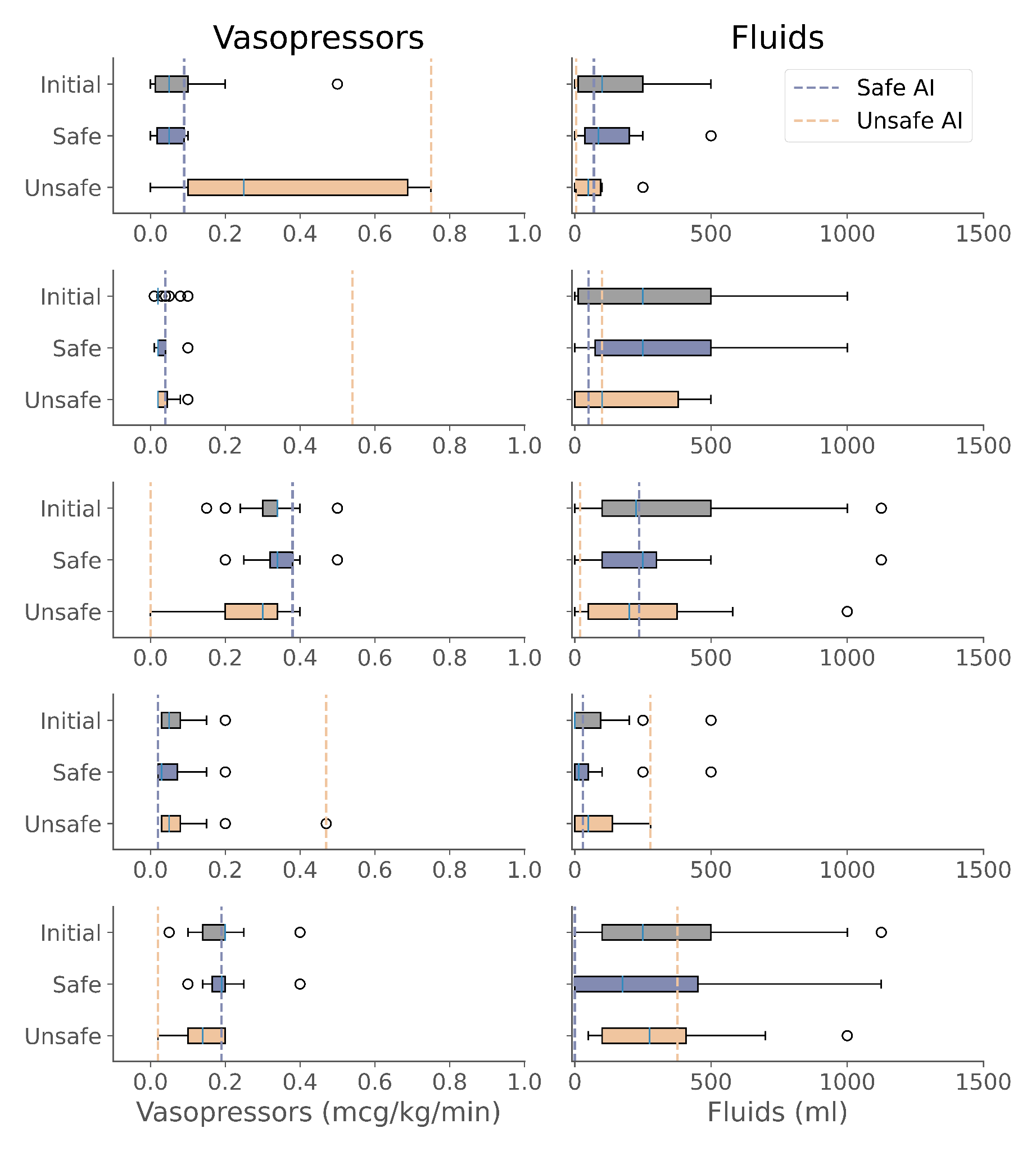

Supplement: S7 Appendix — Extension of Fig 5 showing the dose distribution shifts for all patient scenarios and conditions. (DOCX) [file pdig.0000726.s007.docx]
