# Supplementary material for: Safety of human-AI cooperative decision-making within intensive care: A physical simulation study
Source: PLOS Digit Health. 2025 Feb 24;4(2):e0000726. doi: 10.1371/journal.pdig.0000726 (PMC11849858; doi:10.1371/journal.pdig.0000726)
Supplement: S5 Appendix — Extension of the persuasion coefficient analysis of both safe and unsafe AI recommendations. (DOCX) [file pdig.0000726.s005.docx]

Appendix S5 - Persuasion coefficient of safe and unsafe AI

In an attempt to go further than the binary “accept/reject AI recommendation” outcome, we investigated the impact of safe and unsafe recommendations through their persuasion coefficient. Persuasion coefficients are a common metric in social science to measure the amount of influence a recommendation or advice has had on a decision. It is defined as follows:

[
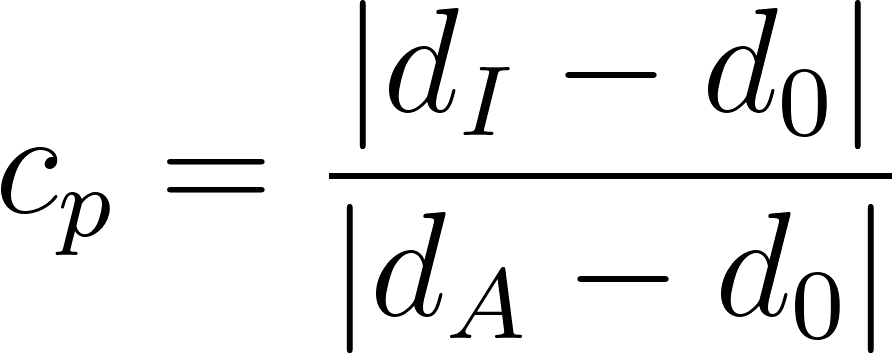
](https://www.codecogs.com/eqnedit.php?latex=c_p%20%3D%20%5Cfrac%7B%7Cd_I%20-%20d_0%7C%7D%7B%7Cd_A%20-%20d_0%7C%7D#0)

Where [
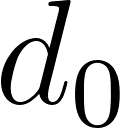
](https://www.codecogs.com/eqnedit.php?latex=d_0#0) is the baseline decision [
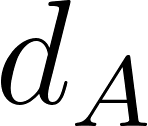
](https://www.codecogs.com/eqnedit.php?latex=d_A#0) the advice/recommendation, and [
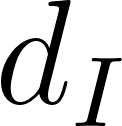
](https://www.codecogs.com/eqnedit.php?latex=d_I#0) is the influenced decision. We calculated this metric for all trials. The below figure shows the resulting distributions. The safe VS unsafe persuasion distributions were not significantly different according to a Wilcoxon rank test.


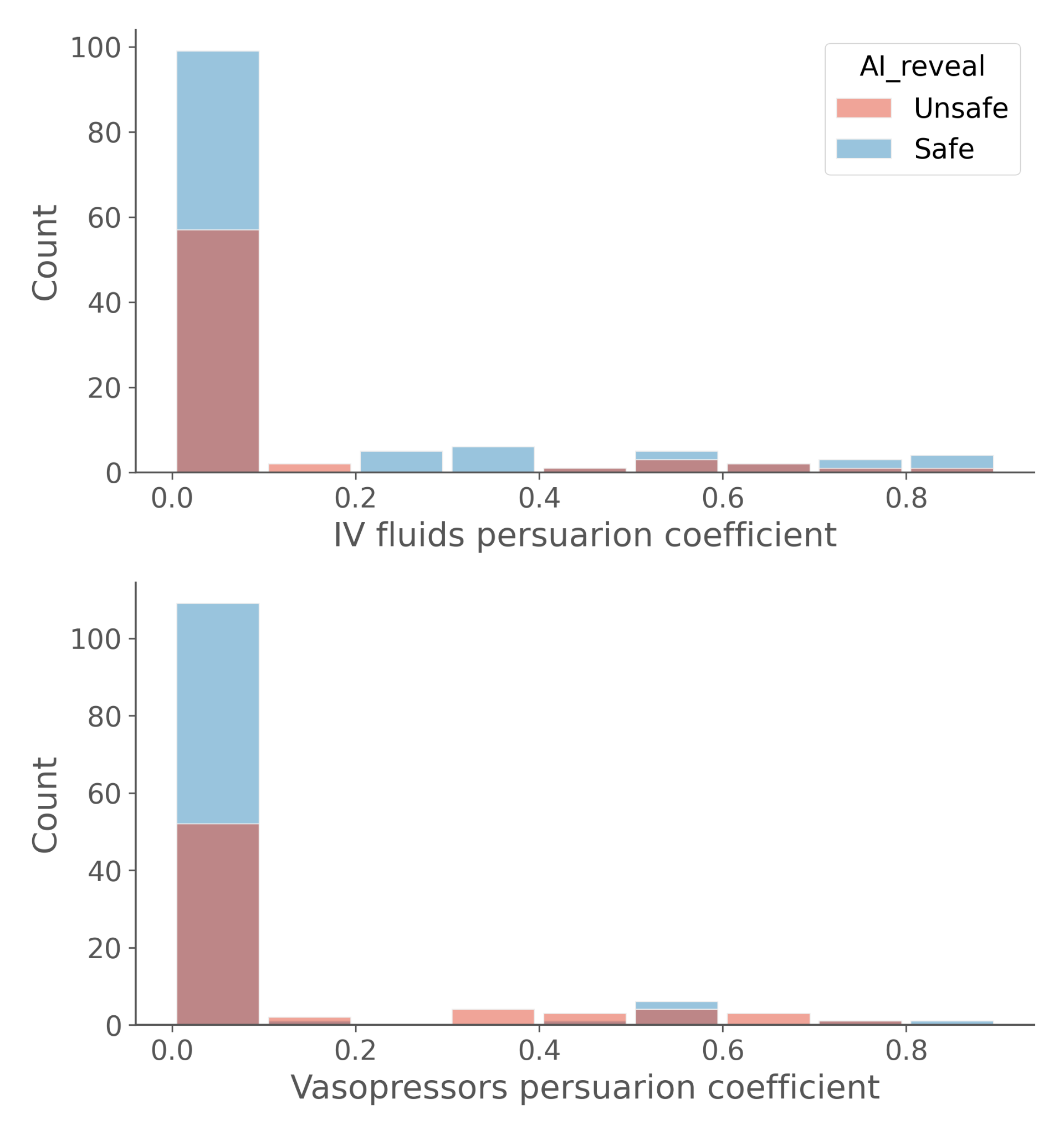


The lack of statistical significance in the difference of persuasion coefficient distributions can be interpreted in different ways: either there is genuinely no signal with this metric, the study does not contain enough data points to make the statistical significance emerge, or the metric is not sensitive enough to make the difference significant.
