# Supplementary material for: Safety of human-AI cooperative decision-making within intensive care: A physical simulation study
Source: PLOS Digit Health. 2025 Feb 24;4(2):e0000726. doi: 10.1371/journal.pdig.0000726 (PMC11849858; doi:10.1371/journal.pdig.0000726)
Supplement: S6 Appendix — Analysis of the link between clinicians giving initial doses far away from the average of their peers and their likelihood of accepting an unsafe AI recommendation. (DOCX) [file pdig.0000726.s006.docx]

Appendix S6 - Initial dose disparity and unsafe AI Acceptance

One of the results of the main paper shows that, even when given the exact same information, clinicians show a wide variety of initial treatment strategies. This appendix looks at whether a subject who takes initial treatment decisions further from the mean of their peers is more likely to accept unsafe AI recommendations. For each patient scenario, the initial discrepancy of a subject was computed as the ratio of their decided treatment divided by the mean of their peers for the same scenario. These proportion were then averaged across all patient scenarios to get an overall initial discrepancy score for each subject. The following figure shows a scatter plot of this initial discrepancy score against the proportion of reflected unsafe AI recommendations for each subject:


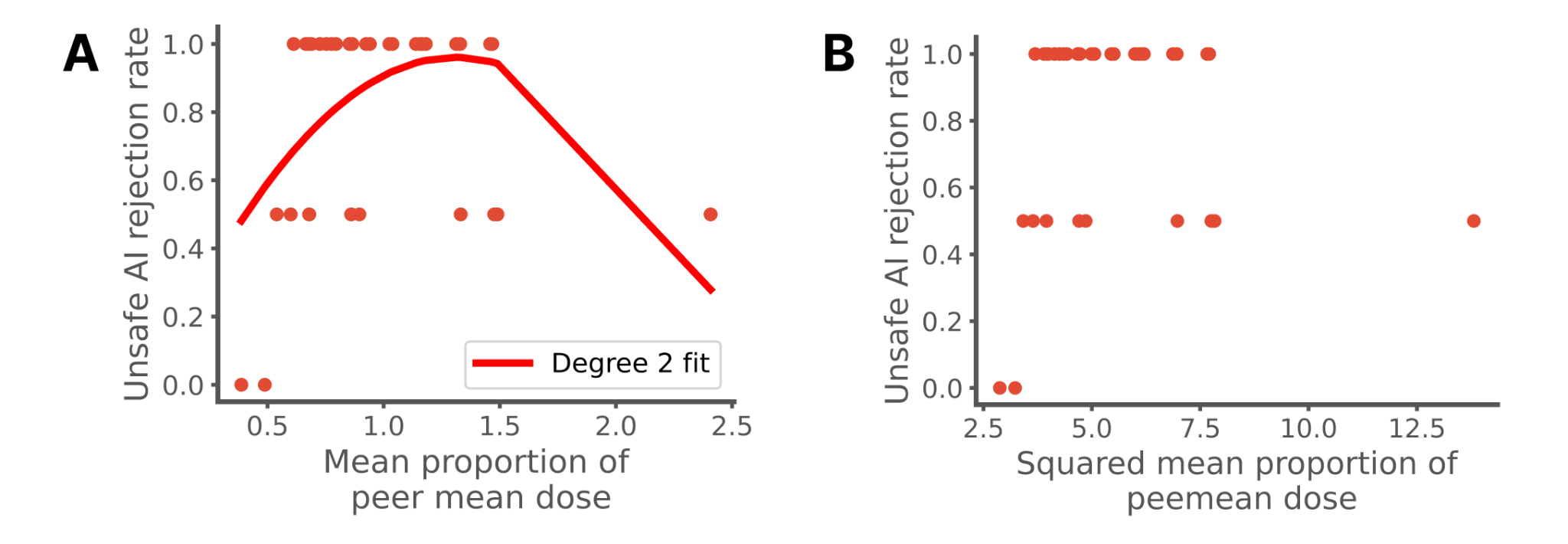


The above figure shows a degree two polynomial fit of the link between the initial discrepancy score, and the proportion of rejected unsafe AI recommendations. While it seems like subjects who take initial decisions furthest from the experimental human consensus are most likely to not reject unsafe AI recommcndations, the second-order correlation was not statistically significant (Person’s correlation test on the centered square of the quantity).
