# Supplementary material for: Safety of human-AI cooperative decision-making within intensive care: A physical simulation study
Source: PLOS Digit Health. 2025 Feb 24;4(2):e0000726. doi: 10.1371/journal.pdig.0000726 (PMC11849858; doi:10.1371/journal.pdig.0000726)

Appendix S8 - Eye-tracking metrics of cognitive load

We looked at proxies for cognitive load through eye-tracking as seen in the literature,^39,40^ and compared them with difficulty scores for each case assigned by the clinicians in the author team. We did not find any significant correlation here.


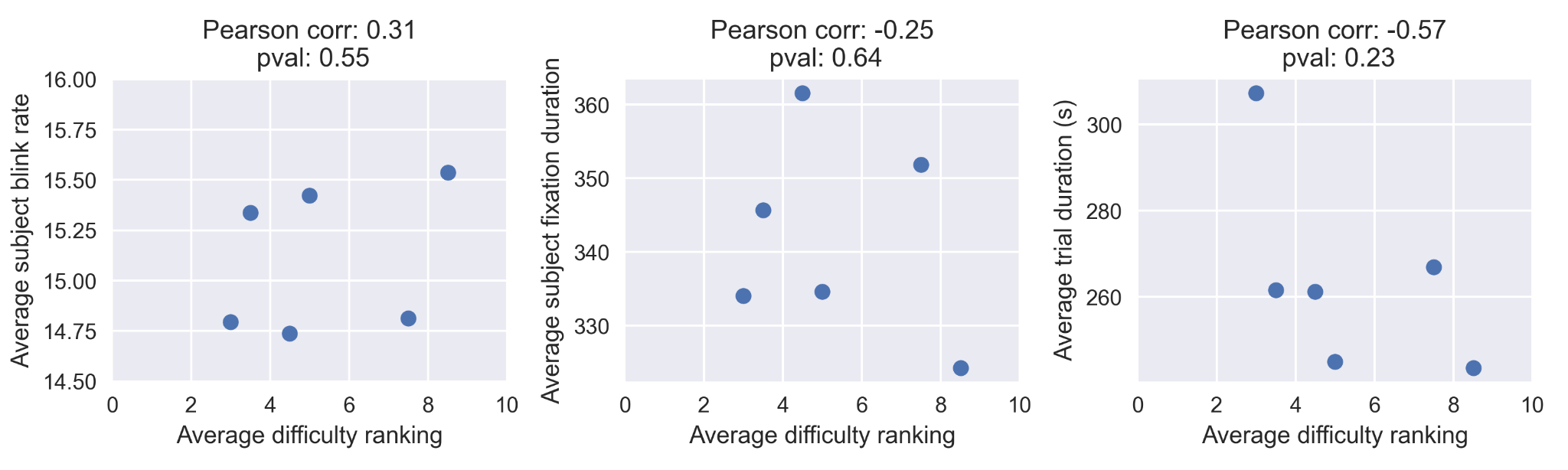

Supplement: S8 Appendix — Graphs of eye-tracking based cognitive load metrics. (DOCX) [file pdig.0000726.s008.docx]
