# Supplementary material for: Safety of human-AI cooperative decision-making within intensive care: A physical simulation study
Source: PLOS Digit Health. 2025 Feb 24;4(2):e0000726. doi: 10.1371/journal.pdig.0000726 (PMC11849858; doi:10.1371/journal.pdig.0000726)
Supplement: Attachment — Submitted filename: ResponseToReviewers.pdf [file pdig.0000726.s010.pdf]

Response to reviewers

Reviewer #1

The research on AI and human interactions in acute healthcare settings is highly valuable, enhancing our understanding of how AI-based clinical decision systems impact healthcare workers and physicians' decision-making processes. The detailed description of the work, along with the comprehensive appendices, greatly facilitates the reproducibility and appraisal of the research by other scholars.

However, the layout of the manuscript could benefit from further refinement. I recommend that the authors have the manuscript reviewed by proof readers not involved in the work to ensure that the text flows coherently for the average reader.
Additionally, the recommendations and lessons drawn from the study should be clarified and distinctly separated from the discussion section. Including a flow chart or a recommendation box could effectively address this issue.

We thank the reviewer for their interest in the work and for recognising its value. The manuscript has now undergone several proofreads by people outside the authorship list, making it more approachable to the average reader. Changes include a new order of sections (Methods went from the end to between Introduction and Results), extension of the context behind certain notions in the introduction, further justification of design choices in Methods, and an enriched Discussion section. To clarify the findings of the work, they are now clearly outlined at the beginning of the Discussion section as bullet points.

Reviewer #2

Dear Editor-in-Chief, Dear Authors,

I am writing to provide my review of the manuscript titled "(Mis)leading the doctor: safety of human-AI cooperative decision-making in hospital settings" authored by Festor et al., which was submitted for consideration in PLOS Digital Health. I have thoroughly reviewed the manuscript, and I am pleased to share my insights and evaluation with you.
I would like to thank the authors for their submission. Trying to understand the human-AI interaction, especially in such a situations like ICU environment is very critical for the efficacy and safety of healthcare.

In general, I still find the article somewhat confusing, mainly due to a bit off order of chapters. It would be important for instance to read the methods before the results to really understand what this is all about. Also, I would suggest authors to introduce the human decision limitations in the introduction. Otherwise criticizing human-AI interaction is only the other side of the coin.

Also, I am a bit confused as to why the least prioritized author is the corresponding author here.
The two last authors jointly supervised the work, as seen by the + next to their names, with their affiliations.

Here are my observations:

Title:

I would suggest the title to be less populistic and more scientific.

Thank you, we have now changed the title to remove the populistic language: "Safety of human-AI cooperative decision-making within intensive care: a physical simulation study"

General:

I suggest you use this order for the headings:
• Abstract
• Introduction
• Materials and Methods
• Results
• Discussionnote
• Conclusions

We have reorganised the manuscript to follow this sequence of headings.

Comments:

113: "However, the final decision remains in human hands." – not always, I would argue Medtronic insulin pump with AI algorithm running the show independently is a high-stake decision making on whether to inject insulin or not. The word "usually" should be added, and the "high-stakes settings" should be defined.
Thank you, this is a good point. We have added "usually" as requested.

115-116: What are the key differences?
We have expanded this point to include references showing AI advice significantly more influent than human advice in both clinical and non-clinical tasks. "and recent work has highlighted differences in the human perception of human vs AI advice ~~.~~ such as the latter being significantly more influential on the final decision (4,5)." l.127-129

117: …requirements… such as?
We have expanded the sentence to include examples of requirements:" In high-risk shared decision-making ~~settings~~ settings, ~~where~~ even non-autonomous AI-decision-support tools have high safety assurance requirements ~~.~~ such as rigorous testing and usage auditing by notified bodies (6, 7)." l.129-132

117: Grammar
124: Grammar
Thank you, these are now fixed.

125: What do you mean by limited availability of human experts?
We mean that specialist clinicians are low in number compared to the general population and already busy with clinical work. For these reasons, it is harder to reach larger numbers of study participants when the participants are clinicians rather than any member of the population.

126-128: You admit that the optimal care is unclear, yet your title and the whole article seems to warn that Human-AI interaction is not perfect. This is confusing, what is perfect and clear then?
We contend that although there is no defined optimal (gold standard) treatment decision, there are known unsafe (wrong) decisions. The paper does not look at the nuances between merely good versus optimal treatment, but rather safe against unsafe by comparing reasonable decisions (which are referred to as "safe") to clearly unsafe ones. A medical justification for the dose classifications for each patient scenario is available in Appendix S3.

123-131: I would add something about the bias where the physicians in unclear high-stakes settings do though trust more on their colleagues, who to be frank are also chaotic, random, prone to mistakes, and are making decisions as "black box". Comparison to standard process is needed in my opinion in the introduction. Also, integrate this part with 140-141.
We have added a line to acknowledge the potential for human decision-making to be "black-box" in nature: "Furthermore, the post-hoc rationalisations of human decision-makers can sometimes be "black-box" in nature, just as AI decisions can be (11)." l.141-143

143: ecologically? What do you mean?
In this context, an ecologically valid experiment refers to an experiment happening in near-real conditions. In the context of ICU, ecological validity contrasts a computer-based vignette-type experiment against an experiment run in a physical ICU simulation suite (based in the hospital with real monitors, displaying continuous data with real alarms and also interaction between members of the clinical team in real time).

162-175 this belongs to Materials and Methods, not here, and can be ignored once the order is correct
We have now reorganised the paper to insert ‘Methods’ between ‘Introduction’ and ‘Results’. We have deleted the small methods summary at the beginning of the ‘Results’ section but kept a few words at the end of the introduction to maintain a good narrative flow of the manuscript.

180: grammar
Fixed, thank you.

182: which were safe / unsafe?
The order of trials is given in Methods and the full trial matrix is available in the supplementary material.

[Sentence from Methods] ”Each physician experienced four safe trials, one unsafe and one “challenged” unsafe in a pseudo-randomised order (see the trial matrix in Appendix S4).” l.250-252

255: surely “confirm” is too strong word here?
This sentence was in an old block which has been replaced by bullet points summarising the study outcomes at the beginning of the Discussion. We added the nuance from our original phrasing which now reads: ”

- AI advice alters treatment decisions and therefore patient care;

” l.469

274: I understood quite major part of the subjects failed to get their eye tracking data collected? That’s a limitation
Indeed, we now mention this in the appropriate section: ” In terms of other data collection inefficiencies, eye-tracking technology fails to capture pupil position for some subjects thus limiting the statistical power of gaze analysis.” l.581-583

301: RL-based?
Apologies, wWe forgot to expand this, and it is now fixed (RL = Reinforcement Learning)

395: Likert scale data analysis?
The Likert scale is a well-known tool in psychometrics to measure opinions in survey research. We have now added a reference to [Joshi et al. 2015] in the sentence.

426-428: this belongs to discussion not here
Indeed, we moved it to a more appropriate place in the Discussion section (see l.TODO).

433-438: no verbs => make a bullet points or a list of these 4 explanations
To help with flow, we restructured this passage as an enumeration and moved the sentences around to improve clarity.

Results:
It would great to summarize results of primary and secondary objectives, also in abstract

We put a list of bullet point conclusions at the beginning of the discussion to summarise the outcomes of our primary and secondary hypotheses, following requests from multiple reviewers who suggested this might increase clarity

Other:
Limitations of “physical simulation suite” vs real world testing? The data is surely not same as from the real world settings.
Also, study was rather small so it is a limitation.
We agree on the limitations of the simulation facility and have added to our limitations section to make this clearer. We have also made the acknowledgement about sample size limitation more prominent: ” While the data is not the same as might be obtained from real-world settings, it is nonetheless far higher fidelity than comparable vignette studies. Moreover, while running the experiment in a simulation suite increases the ecological validity of the results, it also makes recruitment harder as physicians must physically attend (compared to vignettes which can be performed remotely over the internet).” l.577-581

Also, it was performed only in one(?) ICU in UK so no variation between different ICUs and/or countries / cultures can be explored.
We have added a mention to this in the discussion:” This study was run in one simulation suite in a modern university hospital which, although providing a controlled and realistic setting, may not fully capture the diversity and nuances present in different hospital environments.” l.583-585

Figures:
Fig1 make all A/B/C letters either capital or not, now both
We moved all subfigure labels to lowercase for consistency (see Fig 3).

Fig1: B. What does “Override AI if automatically administered?” mean? I am not sure whether automatically means = before asking AI or similar?
This label refers to the following paragraph of the Methods section:
”Finally, physicians were asked whether or not they would stop the AI recommendation if it were to be automatically administered to the patient. This question was intended to nuance the agreement prompt and identify situations where a participant might disagree with an AI recommendation but not necessarily consider it a threat to patient safety. Participants were clearly introduced to the nuance between these two questions in the pre-experiment briefing.
” l.235-240
Clinicians answered whether or not they would have stopped the AI recommendation if it were to be automatically administered to the patient, like an automatic injection pump.

Fig1: what is “April”?
April tags are the QR codes in the pictures used by the eye-tracking software to identify the position of key regions of interest in the field of view. They inherit their name from the April Robotics Lab in the University of Michigan where they were created.

Fig2 A. What are SHO and SpR?
These are UK clinician grades, we now expand them and specify their link to seniority in the figure legend (with reference to US clinician grades).

Fig3. What is XAI?
We have now expanded the term in the legend, it stands for explainable AI.

Appendix S1:
check grammar (e.g. one parenthesis is missing)
Thank you, this has now been fixed.

Appendix S6:
“Cp” should be introduced in the text. The equation and then the explanation contains both capital and small letters, please unify. Also please check the grammar.

Are there only two colors in the bars, as explained in the label? It seems there are multiple colors used.
We have harmonised the cases of the mathematical symbols in the appendix..
We have improved the main text by giving a description of what this quantity means and how it is calculated: ”To bring more depth to the analysis than a binary outcome, we investigated the extent of AI influence which ~~by its persuasion coefficient as~~ was ~~defined in social sciences .~~ calculated on a continuous scale from 0 (completely ignoring advice) to 1 (completely relying on advice) using the formula [(final estimate—initial estimate) / (advice – initial estimate)] per work by Yaniv et al. (39).” l.386-390

The plots show two distributions of two different colours. Each distribution (the blue and the red) is slightly transparent for the reader to see overlaps. The third colour therefore comes from the overlap of the two distributions.

Reviewer #3

We addressed the very insightful comments from the reviewers to the best of our ability whilst trying to limit a dramatic increase in the manuscript word coun. Please find our point-by point answers below:

Reviewer #3: The title uses the term "(Mis)leading," which can be ambiguous. Also, the title does not specify the context clearly. Mentioning that this study is within "hospital settings" is broad; specifying "intensive care units" would provide more precise context.
Thank you, we have now made this change (title is changed to “*Safety of human-AI cooperative decision-making within intensive care*”).

Thank you, in conjunction with the suggestions from the other reviewers we have now revised the wording in the abstract for clarity and impact.

Lines 112-121: The introduction could benefit from a clearer definition of what constitutes "safe" and "unsafe" AI recommendations earlier in the section. Providing examples or a brief explanation here would help contextualize the study for readers. Also, it lacks depth in discussing previous research specifically focused on AI safety in medical decision-making.
We understand the reviewer’s perspective that more could be brought to contextualise this study. This work being very interdisciplinary, it is grounded in a lot of fields (such as clinical medicine, sociology, behavioural psychology, cognitive psychology, human-computer interaction) which we attempt to touch on in the introduction. Moreover, our desire to make this work accessible to a wide audience ranging from medical professionals to computer scientists, introducing the breadth of science that we build upon is key. We moved the Methods section right after the introduction to contextualise the notion of safe and unsafe recommendations easier.

To help with clarity even further, we give an example of an unsafe recommendation right after we introduce this concept in the first paragraph of the Methods section: “~~Our~~ This study had two primary ~~objective~~ objectives. The first was to measure whether participants were able to detect, and correctly reject, unsafe recommendations from an AI tool (such as dramatically increasing doses of vasopressors to a patient with sepsis related to infected and leaky heart valves and elevated blood pressure) and/or ask for senior help when appropriate. The second was to identify whether or not human intervention could interfere with this interaction and sway clinicians away from their initial decision (potentially toward an unsafe treatment).” l.192-198

Lines 122-131: While the example of cardiovascular management during sepsis is relevant, the rationale for choosing this specific example over others in healthcare is not well justified. Additionally,

the discussion about the slow translation to bedside due to patient safety concerns (line 131) would benefit from more concrete examples or data to support this claim.
We have expanded our introduction of cardiovascular management of sepsis to justify this choice more clearly and thoroughly: ”One such example is cardiovascular management during sepsis where the optimal personalised doses of intravenous (IV) fluids and vasopressors remain unknown (15,16). Their personalisations have been designated as a research priority by the international reference taskforce the “Surviving Sepsis Campaign” (17). Sepsis treatment in intensive care embodies all the characteristics that make healthcare AI both a difficult challenge but also one that could have a significant beneficial patient impact if successful. Sepsis affects up to 50 million people around the world with 20% annually dying. It represents the most common cause of death in the hospital and the most expensive condition treated in hospital (18) . As such, applying AI and data-driven approaches to deliver precision medicine therapy for sepsis is a global and urgent research priority. Yet, the translation to the bedside has been slow due to concerns for patient safety and ~~clinicians’~~ clinician trust in these systems ~~.~~ (19-21). ” l.144-156

Lines 156-160: The explanation of the observational study is clear. Nonetheless, it would be beneficial to include more information on how the AI recommendations were generated and validated.
We have now moved the ‘Methods’ section after the ‘Introduction’ to explain all the key study details much earlier in the manuscript.

We explain how the recommendations were generated in the Methods section: ”All AI recommendations were synthetically generated by the research team to ensure a standardised experimental format (i.e. they were not from a real AI system). The definition of unsafe recommendations was based on extreme under- or over-dosing of fluid and/or vasopressor as per previous work ~~.~~ (13).” l.263-266

” The patient scenarios, including the AI recommendations, were piloted with specialist clinicians (not enrolled in this experiment) within the Imperial College Critical Care research group who validated their credibility.” l.248-250

And give a framework for researchers willing to reproduce a similar study in a different field: ” For researchers seeking to evaluate safe and unsafe recommendations similarly, we recommend grounding the choice of criteria for decision safety in a combination of legal documentation (i.e. which decision could make ~~can be evaluated through eye-tracking proxies . While these~~ the user liable), and/or opinions from ~~metrics did not show significant correlations with scenario difficulty (see Appendix S7). Fig 3 shows that recommendation safety impacted the number of fixations on the screen, indicative of~~ a panel ~~higher mental load. The question~~ of domain experts ~~task load on clinicians in~~ (in this case, experienced intensivists) as done in (1).” l.616-623

Lines 161-165: The study's primary aim is clear. However, the secondary objectives, particularly the quantification of shifts in doses and gaze patterns, seem less central to the main safety concern.
The conclusions on dose shifts and eye-tracking are indeed less central to this study. We expand on them in greater detail in a sister paper recently accepted and include only details specifically related to safe vs unsafe recommendations here. We have now added this to the main text: ”We explore eye-tracking data and explainable AI questions deeper in a related/sister manuscript (34).
” l. 186-187

Lines 186-190: You must include more statistical details, such as confidence intervals for the proportions reported, to give a clearer picture of the variability in the data. The statistical significance of the results (p<0.0001) is strong, but the discussion on the non-significant trend for junior vs. senior physicians (lines 191-196) is confusing. It would be beneficial to clarify what these non-significant trends imply and how they might still be relevant. Including qualitative data or direct quotes from participants could provide deeper insights.

We thank the reviewer for their feedback. We have now added 95% confidence intervals for each proportion in the Results section (eg 83±4%, 95% CI: 0.75 - 0.91). We now also explain how they were obtained in the Methods section: “When reporting proportions, we adopt the following format: ~~“~~ “[mean] ±[~~mean±standard~~ standard ~~error~~ error]%, ~~%”. Anytime~~ 95% CI: [Weld confidence interval]”. Any reference to a standard deviation is explicit.” l.293-295
We do not conclude on non-significant trends to respect standard statistical practices. Unfortunately, we did not record verbal statements from the participants in this study, but only a structured post-experiment questionnaire. More detail on this will be available in a sister manuscript very recently accepted elsewhere (Nagendran et al. NPJ Digital Medicine 2024 – we refer to it several times in this paper now).

Lines 198-201: The mention of the persuasion coefficient from social sciences (line 198) is underexplained.
We have now added a longer description of what the persuasion coefficient is as well as how it has been used in other settings.
”To bring more depth to the analysis than a binary outcome, we investigated the extent of AI influence which ~~by its persuasion coefficient as~~ was ~~defined in social sciences .~~ calculated on a continuous scale from 0 (completely ignoring advice) to 1 (completely relying on advice) using the formula [(final estimate—initial estimate) / (advice – initial estimate)] per work by Yaniv et al. (39).” l.386-390

Lines 202-208: The findings on the increase in senior/second opinion requests are insightful, but the connection to overall AI safety could be better articulated. How does this behavior impact patient outcomes or decision-making quality?
Our interpretation is that escalated recommendations need a second opinion to be stopped (no matter their safety). We now clarified this in the Discussion section: “Similarly, high-level supervision of junior clinicians using these tools would ensure constant availability of a second opinion to discuss the system’s output in cases where there might be disagreement.
” l.486-488

Lines 210-217: The use of gaze fixation data (lines 212-217) is innovative, but the implications of these findings are not fully explored. How do these gaze patterns correlate with decision accuracy or safety?
We expand on the link between gaze fixation data and decision safety in the Discussion section, as well as reference our sister paper that looks at this side of the experiment in greater depth: “we also observed that physicians did not rely more on AI explanations in the unsafe scenarios calling into question the use of explanations as a mitigation strategy for unsafe AI. Nor did physicians devote significantly more attention to looking back at the ‘traditional’ (non-AI based) clinical data after seeing an unsafe AI recommendation to understand why the recommendation might be unsafe (i.e. there was no outward evidence of a desire to ‘debug’ the unsafe AI recommendation). The eye-tracking and explainability aspects of this experiment are discussed in greater depth in a sister manuscript (34)” l.524-531

Lines 228-238: The analysis of AI recommendation influence on prescriptions (lines 228-238) would benefit from a more detailed discussion on why safe recommendations had a different influence compared to unsafe ones. What factors contributed to these differences?
We have added some more discussion on the potential reasons for different influences from safe and unsafe AI recommendations. “This difference can be attributed to the nature of unsafe AI suggestions, which involve under- or over-dosing of one or both drugs. The larger discrepancy between unsafe AI suggestions and physicians' typical practices likely contributes to the reduced influence of unsafe recommendations.” l.432-435

Lines 240-248: The "challenge" section with the bedside nurse (lines 240-248) provides valuable insights into human-to-human interactions influencing AI decisions. However, the sample size (only two participants swayed) is too small to draw any robust conclusions. This limitation should be acknowledged more explicitly.

We have now made this limitation more explicit by adding: "Finally, the results from this study demonstrate the existence of human-human influence, within the context of interacting with AI, but the sample size limits the capacity to accurately estimate the likelihood and significance of the phenomenon or generalise it to different settings." l.590-593

Lines 255-263: The discussion would benefit from a more in-depth exploration of the implications of these findings for clinical practice. For example, what specific training or support might help clinicians better evaluate AI recommendations? Engage more thoroughly with existing literature. For example, discussing Hasan et al. (2024) would contextualize your findings within the broader field of ethical considerations in healthcare practice. https://doi.org/10.1186/s12910-024-01062-8
Integrate a section discussing the ethical implications of using AI in clinical decision-making, drawing parallels with the concerns raised in the pharmacy practice study. Recommend enhanced education and training for healthcare professionals to improve their understanding of AI, addressing both ethical concerns and practical challenges.
Thank you for pointing our attention towards this interesting paper. We have now added an extended discussion of the ethical implications of using AI in medical decision making in the Discussion section, including a reference to this paper. "Our findings highlight important implications for clinical practice that warrant further exploration. Recent work by Hasan et al. examines the ethical considerations of AI in pharmacy practice and reveals similar themes around patient autonomy, data protection, and the need for regulatory frameworks (43). Their study found widespread concerns about patient data privacy, cybersecurity threats, and lack of legal regulation among pharmacy professionals. To address these issues in clinical settings, we recommend developing comprehensive education and training programs for healthcare professionals that encompass both technical skills and ethical considerations. Such programs could include AI literacy modules, case studies on responsible AI integration, ethical frameworks for evaluating AI recommendations, and hands-on training with AI tools. Healthcare institutions should also consider establishing AI ethics committees to provide ongoing guidance and oversight.

The ethical implications of using AI in clinical decision-making are profound. While AI has the potential to enhance diagnostic accuracy and treatment optimization, it also raises concerns about the erosion of human judgement and the patient-provider relationship. To mitigate these risks, we advocate for a human-centred approach to AI integration that preserves clinician autonomy while leveraging AI as a supportive tool. By proactively addressing these ethical and practical challenges, healthcare systems can work towards responsible AI adoption that enhances rather than compromises the quality and humanity of patient care.
" l.499-518

Lines 255-256: The assertion that AI recommendations "can influence clinician behaviour" is overly simplistic. This is not a novel insight, as the influence of AI on decision-making is a well-documented phenomenon across various fields. The discussion would benefit from a more nuanced exploration of how different types of AI recommendations influence clinician behavior in specific contexts.
We have made this section more comprehensive by referring to various other studies that document the effects of AI recommendations in other medical contexts (for example radiology): "The influence of AI recommendations on clinical judgement has ~~already~~ been studied previously in vignette-type ~~experiments~~ experiments. ~~.~~ In tasks with a gold standard to compare against like medical imaging, AI advice has been shown to impact clinical decisions differently depending on its quality and the level of expertise of the clinical user. In this context, explainability did not help physicians identify biased models (45,46). Similarly, even in settings with no gold standard like cardiovascular management of sepsis in intensive care, AI advice has been shown to significantly shift final treatment decisions in a vignette setting (4)." l.533-540

Lines 258-263: The emphasis on the need for educating clinical teams is appropriate but not sufficiently developed. The statement is vague about the specifics of the educational content and methods. Moreover, the discussion should address the potential challenges and barriers to effective education, such as time constraints, resource availability, and varying levels of AI literacy among clinicians.

We agree with the reviewer that educating clinical teams is important and needs much more research. As many questions remain unanswered, and education was outside of the remit of this research, we feel it would be speculative to go into too much detail at this time. We have expanded the discussion as follows: "We hypothesise that the human influence towards unsafe recommendations could have been avoided by a small briefing on the key points touched on by the bedside nurse: technology benefits, limitations, and legal paradigms. Domain experts should explore how to provide effective education given the context of limited time, resources, and varying AI literacy among clinicians." l.481-485

Lines 266-270: The claim that the study's design allows for the observation of human-human dynamics in AI interaction lacks empirical support. The authors should provide more detailed evidence from their data on how these dynamics manifest and their impact on decision-making. Additionally, more clarity is needed on how these observations can be generalized beyond the study's simulated environment.
We acknowledge that the primary focus of our study was not on human-human interactions in the context of AI-driven decision support tools but instead on how the individual practitioner interacts with the AI. However, as we were already conducting the experiment in a high-fidelity simulation suite, a logical exploratory step that seemed worthy of investigating was how a second human (in this case the bedside nurse) might influence the subjects. It is likely that our observations cannot necessarily be generalised beyond this study to other specialties or settings. For example, the impact of human-human interactions within a complex cancer imaging MDT (multi-disciplinary team) conference where AI-suggested radiological diagnoses are being discussed might be very different given that there is much more time available for discussion and the decisions may be more nuanced. The field of multi-directional AI-humans interactions would need further dedicated studies focused on this human-human influence element . We added the following text to the discussion: "Finally, the results from this study demonstrate the existence of human-human influence, within the context of interacting with AI, but the sample size limits the capacity to accurately estimate the likelihood and significance of the phenomenon or generalise it to different settings. The human-human influence in a human-AI team remains an open research field, especially for multi-disciplinary teams." l.590-594

Lines 271-273: The authors should discuss the limitations of simulation suite approach more thoroughly. Simulation environments cannot fully replicate the complexities and pressures of real clinical settings. The discussion should address the potential biases introduced by the simulated environment and how they might affect the study's findings.
Thank you for this important suggestion. We have now added: " Although the simulation suite replicates many of the features of the clinical environment (including real bedside monitors displaying data in real time, with working alarms and some human interactions) it cannot completely replicate the competing priorities and time-pressured clinical environment of a real intensive care unit. Additionally, we were not able to replicate the dynamic sequential assessment/ treatment decisions provided over time, as we asked subjects for a single decision on each of six separate patients. Assessing multiple decision points in each scenario would have been more reflective of real clinical practice, but would have created an exponential number of potential scenarios, and therefore led to less similar situations to compare in the statistical analysis." l.568-576

Lines 276-283: The manuscript could be strengthened by discussing the limitations of eye-tracking as a measure of cognitive load and suggesting complementary methods that could be used in future research. The authors should explore alternative explanations for this behavior, such as inherent human attention biases toward negative stimuli. Additionally, the practical implications of these findings for clinical practice and AI system design are not adequately addressed. Also, the authors should delve deeper into why this might be the case. Possible factors could include mistrust in AI explanations, lack of clarity in the explanations provided, or cognitive overload. The discussion should propose potential solutions or areas for further research to enhance the usability and trustworthiness of AI explanations.
We agree with the reviewer that the implications, strengths and limitations of eye-tracking are important. This is comprehensively discussed in a sister manuscript that has been accepted for publication in another journal and which we reference here:

1. Nagendran M, Festor P, Komorowski M, Gordon AC, Faisal AA. Eye tracking insights into physician behaviour with safe and unsafe explainable AI recommendations. NPJ Digital Medicine. 2024; *accepted for publication*.

We directly reference this linked manuscript at the end of the introduction: " We explore eye-tracking data and explainable AI questions deeper in a related/sister manuscript (34)." l.186-187

and in the discussion: " The eye-tracking and explainability aspects of this experiment are discussed in greater depth in a sister manuscript (34).
" l.529-531

Lines 296-309: It would be helpful to discuss potential strategies to address these limitations in future studies. Also, prescribing hourly fluid and vasopressor doses is not typical practice for intensive care physicians and raises concerns about the ecological validity of the study.

Thank you for this comment. We state the limitation of hourly prescriptions in the Discussion section: "The ~~However, the~~ limitations of the study should also be acknowledged. First, ~~as raised by many physicians during the initial briefing,~~ prescribing hourly fluid and vasopressor doses directly is unusual for intensive care physicians who typically indicate blood pressure ~~(and other parameters)~~ or urine output targets and let the bedside nurse titrate the actual doses within a reasonable range to reach the set targets. However, direct drug dose recommendation has become the standard for reinforcement learning ~~RL-based~~ (RL)-based decision support tools in healthcare (48,49). ~~for sepsis management .~~ While clinicians do not usually work directly with drug doses in their current practice, the AI systems they will encounter in their practice likely will and so our study better replicates the actual context in which clinicians will interact with AI prescription decision support ~~tools.~~ tools in the ICU." l.556-560

Lines 311-331: The critique of the definitions of safe and unsafe recommendations is crucial. The discussion stops short of proposing concrete ways to address this issue. The authors should consider suggesting standardized guidelines or frameworks for defining and evaluating AI recommendations in clinical settings. Furthermore, the concept of the "mysterious oracle dilemma" is intriguing and warrants deeper exploration. How can clinicians balance the potential benefits of novel AI recommendations with the risks of occasional suboptimal outcomes?

Thank you for raising these key points. First, we expanded the discussion to suggest ways for other researchers to come up with criteria for safe and unsafe decisions based on the strategy we followed: "For researchers seeking to evaluate safe and unsafe recommendations similarly, we recommend grounding the choice of criteria for decision safety in a combination of legal documentation (i.e. which decision could make ~~can be evaluated through eye-tracking proxies . While these~~ the user liable), and/or opinions from ~~metrics did not show significant correlations with scenario difficulty (see Appendix S7). Fig 3 shows that recommendation safety impacted the number of fixations on the screen, indicative of~~ a panel ~~higher mental load. The question~~ of domain experts ~~task load on clinicians in~~ (in this case, experienced intensivists) as done in (1)." l.616-623

Second, we wholeheartedly agree on the importance of the mysterious oracle dilemma, to the extent that we would like to formalise its definition and implications, which likely deserve to be expanded in a separate manuscript in the future. We expanded the discussion to point towards relevant work from the philosophical literature and indicate it as an interesting direction to be pursued: " This dilemma relates to philosophical discussions on epistemic authority and testimonial knowledge (52,53), and deserves more discussion in future works." l.611-613

Lines 333-342: The authors should provide more specific recommendations for how their findings can inform regulatory practices. Additionally, the potential role of generative AI in clinical settings is briefly mentioned. This is a critical area that deserves more attention, particularly in light of recent advances in AI technology.

We expanded our discussion to clarify the link between our work and the evolving regulatory landscape and generative AI: "As regulators ~~push toward requiring~~ require clear intended purpose statements for software as medical devices ~~,~~ (57), our high-fidelity ~~eye-tracking~~ eye-tracking-based ~~based~~ approach to evaluating an AI-driven decision support tool serves as a basis for promoting the

generation of safety evidence. evidence, with a particular emphasis on ecologically valid settings. This work also provides pointers on how medical education could tailor for the increasing use of AI technologies in medical decision-making. Furthermore, the recent rise in popularity of generative AI (most notably as large language models) highlights the safety concerns of hallucinatory outputs that might be acted upon in a clinical setting and bring harm to patients . (58,59). The unsafe AI recommendations in this work share characteristics with generative AI hallucinations: they are presented to users with no warning and as much confidence as safe recommendations. It is likely that an AI system that shows overall superhuman performance in a given task will still show lower-quality performance in some specific cases . (60). Solutions such as uncertainty-aware models or explainable AI might help users differentiate between well-informed recommendations and flawed calls . (40,61,62). The human-AI interactions at the bedside, with a particular focus on high-pressure decision-making, would also help to accelerate the safe translation of AI-based decision support tools to the bedside" l.624-650

Lines 346-357: In conclusion consider adding a forward-looking statement on the next steps for research in this area and how the findings could influence policy and practice in AI deployment in healthcare.
As described above, we have included more detail about future work needed in this area in the discussion. In order to avoid too much speculation in the conclusion, we kept our statements focussed on our results and have suggested some future directions of work in this area: "Uncertainty awareness, novel forms of AI interpretability and a better understanding of human-human interactions (i.e. team decisions) in the context of AI-driven decision support will help not only with assur assuring safety from a regulatory perspective but also in fostering confidence and approval trust from physician clinician end-users end-users." l.660-664

Lines 360-372: The study design briefing and pre-experiment questionnaire details (line 371) are relegated to the supplementary material. Including a summary of key questions and their relevance to the study will strengthen this section.
We expanded this section to contextualise the onboarding phase better. The details remain available in the supplementary appendix to maintain good flow while keeping the information easy for the reader to find: "The questionnaire recording recorded their demographics and (age, gender, years of experience prior),prior experience with AI, as well as attitude towards AI (see through questions on their perception of AI benefits/ issues.See Supplementary Appendix S1 for the full content of the briefing and questionnaire). questionnaire." l.204-208

Lines 379-418: In the description of the simulation scenarios (lines 379-418), the justification for the specific number of scenarios (six) and the mix of safe, unsafe, and "challenged" unsafe trials could be better explained. Why was this mix chosen, and how does it ensure robust data collection?
We have now expanded our justification for the number of scenarios per participant in the Methods section: "Physicians experienced more safe than unsafe trials to maintain some form of trust in the system. The There only was no time pressure on decision-making came from the physicians to make the their decisions: need to fit six scenarios in early dry-runs we estimated 7 minutes per trial, so we fitted 6 trials in the 45 minutes. minutes allocated to the experiment. Actual trial length depended on the speed of individual physicians." l.255-260

Lines 421-431: The synthetic generation of AI recommendations (lines 421-431) ensures standardization but also raises concerns about ecological validity. How realistic were these recommendations perceived by the participants, and how might this affect the study's conclusions?
The study participants did agree with the majority of safe recommendations, hence finding them realistic. This is presented in our results (Figure 3.A). Unsafe recommendations were refined by our expert panel to appear unacceptable by design. We agree that a higher resolution in the realistic to unrealistic recommendation spectrum would have been useful. However, more granularity would have reduced the statistical power of the study.

Lines 451-485: In the use of eye-tracking technology (lines 451-485), the high exclusion rate (only 19/38 physicians) due to calibration issues is problematic. This could introduce bias, and the impact of this exclusion should be discussed more thoroughly.
We agree that there is potential for bias due to the high exclusion rate although there was no obvious difference of this observed (felt to be missing at random in all likelihood). We have made this more explicit in the text: "Subjects were ~~negligible~~ eligible for eye-tracking data collection according to the following inclusion ~~criterion:~~ criteria: the ~~PupilLabs~~ Pupil Labs glasses were able to detect their pupils, the calibration step was passed successfully, and the glasses-laptop connection was not lost during the experiment. In total, 19/38 physicians passed the calibration exercises and had their gaze-based attention data collected. There was no evidence that this ~~broke down across~~ data loss led to any ~~obvious subgroups.~~ inclusion bias. 13/19 male doctors had gaze data (68% male compared to 66% male in the overall sample). 6/19 senior doctors had gaze data (32% senior compared to 55% senior in the overall sample)." l.315-322

As well as: "In terms of other data collection inefficiencies, eye-tracking technology fails to capture pupil position for some subjects thus limiting the statistical power of gaze analysis." l.581-583

Lines 487-492: The recruitment strategy (lines 487-492) seems sound, but the use of convenience sampling could lead to selection bias. More details on how this was mitigated or its potential impact on the findings would be beneficial.
This was not specifically mitigated against. It was difficult to recruit ICU clinicians for the study given their frequent out of hours shifts and heavy clinical commitments. We therefore felt that convenience sampling was a reasonable trade-off to enabling us to meet the sample size requireement. We have added this limitation to the discussion section: "Potential biases from the use of convenience sampling were not particularly mitigated against, we thought it was a good trade-off between clinicians' low availability (frequent out of hours shifts and heavy clinical commitments) and the study's rectuitment target" l.586-588

Technical Corrections:
Line 117: "understanding" should be capitalized as it starts a new sentence.
Line 120: Replace a comma after "yet" for better readability.
Thank you, these are now fixed.

Lines 144-147: The word "simulation" is repeated; consider rephrasing for clarity.
We edited the sentence to improve clarity: ". Simulation exercises have ~~This is a tool which has~~ historically been used as a widely accepted training tool for both modelling high-fidelity situations and capturing patterns of human ~~behaviour~~ behaviour. They ~~with simulation~~ now ~~forming~~ form a core part of medical training ~~.~~ (29)." l. 170-173

Line 244: "bedside nurse did not sway the physician's decision" - consider rephrasing.
We have rephrased to: "the bedside nurse did not ~~sway~~ change the ~~physician's~~ decision of the physician" l.447-448

Figures and Tables:
Ensure all figures and tables are clearly labeled and referenced in the text. The legends should provide sufficient detail to understand the content without referring back to the main text.
All figures are referenced in the main text. Edits have been made to improve the clarity of the legends:

- Fig.1 now mentions limitations and points to their discussion in the main text;
- Fig.2 now mentions the sample size of the study;
- Fig.3 clarifies the statistical method for the p-value in the plots;
- Fig.4 is now further discussed in the Results section and is associated with a supplementary figure in the appendices;
- Fig.5 now has more details in its legend to improve the clarity and implications of the plots.

Fig 1: The experimental design lacks clarity in conveying the simulation's realism and its applicability to real-world settings. The choice of using a mannequin and eye-tracking technology does not sufficiently address potential limitations such as the lack of dynamic interaction with a real patient and the artificiality of the environment, which could influence clinician behavior differently compared to a real ICU setting.
We have expanded the figure legend to point at the discussions of the setup in the main text: "**Fig 1: Experimental design – A.** ~~Photo~~ Visual representation of the simulation suite ~~with:~~ and overall summary of experimental design. A full description and rationale for the methods chosen including the realism limitation of the environment is given in the main text.: (1) Bedside monitor (2) […]"

Fig 2: The sample size is not mentioned, which is critical for assessing the study's statistical power and the generalizability of the findings. Additionally, the proportion of participants previously involved in AI research is noted, but the potential impact of their prior experience on study outcomes is not explored.
We added the sample size in the figure legend (n=38). The main text (Results section) answers the reviewer's question on the impact of prior AI experience on study outcomes: "While we recorded previous experience with AI as an independent variable, our analyses did not find any impact on the primary outcomes of this study." l.452-454

Fig 3: The interpretation is overly simplistic. The discussion should provide a more thorough analysis of why clinicians might stop safe recommendations or escalate unsafe ones.
The statistical significance (p-values) needs more context to understand the practical relevance of these findings.
Figure 3 reports the proportions of study participants who stopped or "escalated" cases in different scenarios. Our study did not collect information about the reasons why such decisions were made and therefore any further analysis of root causes behind decisions would be speculative at this time. This would require further qualitative studies.
We have now added more detail about p-values in the figure legend: "Z-score Bonferroni-corrected proportion statistical tests: *** $p<0.0001$, ** $p<0.005$, * $p<0.05$, *n.s .* not significant, error bars represent the standard error of the mean ~~(SEM).~~ (SEM), XAI = explainable AI."

Fig 4: The observed variability in clinical practice underscores the complexity of clinical decision-making. However, the figure fails to adequately correlate these variances with AI influence, and the discussion does not sufficiently address how AI might standardize or exacerbate these differences.
This figure reports the variability of the initial decisions to illustrate that this variability exists even when controlling all the input information. This is an important finding in itself. Your point being very interesting, we expanded the results to include the link between giving outlier initial doses and accepting unsafe AI recommendations. We added our results in the main text as well as plots in the supplementary appendices: "We also evaluated the extent to which being far from the human mean dose initially was indicative of not stopping unsafe AI recommendations. While subjects with most unsafe AI misses were further from the mean, there was no statistically significant correlation between the distance to human mean in initial dose and likelihood of accepting an unsafe AI recommendation ~~see~~ (see Supplementary Appendix S8)." l.419-424

Fig 5: The conclusion that unsafe AI recommendations do not always influence the final decision needs more robust evidence. The discussion should delve deeper into the factors that contribute to a physician's decision to seek a second opinion and how this behavior interacts with AI recommendations.
We agree with the reviewer that a greater understanding of expert decision-making in sepsis management in intensive care would be highly valuable. However, as mentioned above, we unfortunately did not collect free-text feedback from clinicians as part of this study. Drawing conclusions on the reasons behind their decisions from our data would therefore be purely speculative.
We have expanded the legend of Figure 5 to improve comprehension: "**Fig 5: Shift analysis –** Distributions of ~~Vasopressors~~ vasopressors dose distribution before and after having seen a safe or unsafe AI recommendation for two patient scenarios across all physicians (left) and only those who

did not ask for ~~senior/second review~~ senior help /second opinion (right). For each group of physicians and patient scenarios, we show the distributions of doses before seeing any recommendation (grey), after seeing a safe recommendation (blue), and after seeing an unsafe one (yellow). The dashed bars represent the values of the safe and unsafe AI recommendations for each patient scenario. Unsafe AI recommendations do not always influence the final decision. Physicians most influenced by unsafe AI recommendations tend to ask for a second opinion, while those who do not ask for help are less influenced by unsafe recommendations. ”

Appendices
The questionnaire lacks depth in exploring potential biases or preconceptions that could influence study outcomes.
We agree with the reviewer’s comment. The post-experiment questionnaire was kept limited in length given the duration of each experiment. The potential biases and preconceptions from participants was not the main focus of the study and would be better investigated formally with a qualitative interview study, which we hope to do in the future.

The nurse challenge scripts does not adequately analyze how these challenges affected clinician decision-making and their interactions with AI recommendations.
The impact of the nurse challenges is reported in the Results section of the main paper, and briefly discuss the significance of this result: “In 95±4% (95% CI: 0.87 - 1.03) of cases (36/38), the verbal input challenge from the bedside nurse did not ~~sway~~ change the ~~physician's~~ decision of the physician to accept or reject the automated application of the AI recommendation. However, two participants (both junior) were persuaded to change their minds from interrupting an unsafe recommendation to accepting it.” l.446-450

And acknowledge the limitations of our sudy on the matter in the discussion: “Finally, the results from this study demonstrate the existence of human-human influence, within the context of interacting with AI, but the sample size limits the capacity to accurately estimate the likelihood and significance of the phenomenon or generalise it to different settings. The human-human influence in a human-AI team remains an open research field, especially for multi-disciplinary teams” l.590-594

The detailed patient scenarios are comprehensive, yet the variability in clinical presentations might introduce uncontrolled variables. The justification for AI actions needs a more rigorous examination to ensure they are perceived as credible by the participants.
This represents a trade-off (comprehensive scenarios spanning several presentations versus variation and reduced standardisation).
We piloted the scenarios including the AI actions with specialist doctors from Imperial College London Critical Care Research group who perceived them as credible. In total, a doxen of intensivits answered whether or not each scenario was realistic, the safe recommendation reasonable and the unsafe unreasonable. They unanimously agreed with the scenarios. We have now added this information to the main text: “The patient scenarios, including the AI recommendations, were piloted with specialist clinicians (not enrolled in this experiment) within the Imperial College Critical Care research group who validated their credibility.” l.248-250

The use of persuasion coefficients requires a more detailed explanation and justification.
The persuasion coefficient as we define it in the paper has been used in social sciences to study the impact of advice on participant decisions (Yaniv et al, Organ Behav Hum Decis Process 2004). We added a clearer explanation of this metric in the manuscript: “To bring more depth to the analysis than a binary outcome, we investigated the extent of AI influence which ~~by its persuasion coefficient as~~ was ~~defined in social sciences .~~ calculated on a continuous scale from 0 (completely ignoring advice) to 1 (completely relying on advice) using the formula [(final estimate—initial estimate) / (advice – initial estimate)] per work by Yaniv et al. (39).” l.386-390

The lack of significant differences between safe and unsafe persuasion distributions should prompt a reevaluation of the metric's sensitivity and applicability.
Thank you for this comment. We added the following discussion on this in the supplementary appendix on the "persuasion coefficient": "The lack of statistical significance in the difference of persuasion coefficient distributions can be interpreted in different ways: either there is genuinely no signal with this metric, the study does not contain enough data points to make the statistical significance emerge, or the metric is not sensitive enough to make the difference significant." (Appendix S6)

The attempt to correlate eye-tracking metrics with cognitive load and case difficulty lacks a significant findings suggests either a limitation in the measurement approach or the need for a more refined analysis.
We respectfully disagree as this suggests either the measurement devices were wrong, or the analysis method was flawed. It is also possible that a lack of statistical significance could reflect the fact that the two analysed quantities may not be correlated.
The eye-tracking measurement device we use is accurate enough to give reliable blink rate and fixation duration numbers. The case-difficulty score is an average of scores given by expert clinicians, which might not be perfect but should be representative of the different cases used. We used a standard method to compute correlation which confirmed that the value was not significant.
The paper does not claim that true cognitive load was not linked to true case difficulty, but simply that our proxies for these two values were not correlated. We acknowledge that these proxies are imperfect and do not make any wider claims than what is supported by our analyses. See: "Another interesting angle for analysis is cognitive load, which can be evaluated through eye-tracking proxies (54,55). These metrics did not show significant correlations with estimated scenario difficulty (see Appendix S7)." l.627-629

Reviewer #4

Reviewer #4: The manuscript presents a study on the interaction between human clinicians and artificial intelligence (AI) decision-support systems in a high-fidelity simulation environment. The study aimed to evaluate the ability of clinicians to detect and reject unsafe AI recommendations in a sepsis management scenario.

The study involved 38 intensive care physicians who participated in a simulated ward round, where they were presented with six patient scenarios and asked to prescribe fluid and vasopressor doses before and after receiving AI recommendations. The AI recommendations were synthetically generated and included safe, unsafe, and "challenged" unsafe scenarios.

I really enjoyed reading the manuscript as its main messages are extremely important for our understanding of implementing AI tools in clinical practice. I only have a few minor suggestions:

1. The authors state that Clinicians did not look back at the original data to "debug" why an unsafe recommendation was given by the AI system. It might be interesting to understand, why clinicians didn't try to debug. Were the participating clinicians interviewed and confronted with this finding afterwards?

We agree with the reviewer that a better understanding of the mechanisms at play in the mind of clinicians when facing AI recommendations would be highly valuable. However, our pre- and post-study questionnaire were kept limited in length given the already long duration of the main study. The potential biases and preconceptions participants may have was not the main focus of the study and would be better investigated formally with a qualitative interview study, which we hope to do in the future.

2. The sentence in line 162 is redundant and could be removed.

This sentence was there to improve the flow of a manuscript that had the Methods section at the end. After feedback from the reviewers, we moved the Methods section back between Introduction and Results and removed this redundant sentence.

3. It seems that the majority of clinicians were male. Did the authors investigate, if gender may have an impact on the results?

We did not specifically investigate the impact of gender on the main experimental conclusions. However, we checked that the gender ratio in our participating population is similar to the national UK intensivist gender ratio, supporting the representative nature of our sample population.

4. From a statistical point of view, the authors used a relatively low number of clinicians/subjects in their study, which is appropriate for a pilot study. What remains unclear to me is, how the number of participating clinicians was estimated, if at all (the authors state in the manuscript that "The number of safe and unsafe scenarios was chosen to balance balance recommendation type and statistical power").

The number of subjects was obtained after power calculations, balancing logistical capabilities and expected results. The power calculation showed that around 40 subjects would be enough to get statistical significance at level 0.05 for a 15% difference between groups in the main study outcome (stopping the AI and/or asking for senior help in safe and unsafe recommendation trial groups).

In order to be representative of a positive projection of AIs in ICUs, we also had the criterion to show participants more safe recommendations than unsafe ones. We considered the trade-off between the number of recorded decisions (subjects times trials per subject), and the minimum statistically significant proportion difference we could observe with a Bonferroni-corrected z-test.
